# Ionizing Radiation Curtails Immunosuppressive Effects From Cancer-Associated Fibroblasts on Dendritic Cells

**DOI:** 10.3389/fimmu.2021.662594

**Published:** 2021-06-09

**Authors:** Rodrigo Berzaghi, Stian Tornaas, Kristin Lode, Turid Hellevik, Inigo Martinez-Zubiaurre

**Affiliations:** ^1^ Department of Clinical Medicine, Faculty of Health Sciences, UiT-The Arctic University of Norway, Tromsø, Norway; ^2^ Department of Radiation Oncology, University Hospital of Northern Norway, Tromsø, Norway

**Keywords:** cancer-associated fibroblasts (CAFs), monocyte-derived DC, immunosuppression, ionizing radiation, radiotherapy, non-small cell lung cancer, tumor microenvironment

## Abstract

Cancer-associated fibroblasts (CAFs) participate actively in tumor development and affect treatment responses, by among other mechanisms, promoting an immunosuppressive tumor microenvironment. In contrast to normal fibroblasts, reactive CAFs secrete a myriad of immunomodulatory soluble factors at high levels, i.e. growth factors, cytokines, and chemokines, which directly influence tumor immunity and inflammation. CAFs have been identified as important players in tumor radioresistance. However, knowledge on the immunomodulatory functions of CAFs during/after radiotherapy is still lacking. In this study, we investigated the effects of ionizing radiation on CAF-mediated regulation of dendritic cells (DCs). CAFs were obtained from freshly operated lung cancer tissues, while DCs were procured from peripheral blood of healthy donors. Experimental settings comprised both co-cultures and incubations with conditioned medium from control and irradiated CAFs. Functional assays to study DC differentiation/activation consisted on cytokine release, expression of cell-surface markers, antigen uptake, migration rates, T cell priming, and DC-signaling analysis. We demonstrate that CAFs induce a tolerogenic phenotype in DCs by promoting down-regulation of: i) signature DC markers (CD14, CD1a, CD209); ii) activation markers (CD80, CD86, CD40, and HLA-DR) and iii) functional properties (migration, antigen uptake, and CD4^+^ T cell priming). Notably, some of these effects were lost in conditioned medium from CAFs irradiated at fractionated medium-dose regimens (3x6 Gy). However, the expression of relevant CAF-derived regulatory agents like thymic stromal lymphopoietin (TSLP) or tryptophan 2,3-dioxygenase (TDO2) was unchanged upon irradiation. This study demonstrates that CAFs interfere with DC immune functions and unveil that certain radiation regimens may reverse CAF-mediated immunosuppressive effects.

## Introduction

Recent studies in both pre-clinical and clinical settings have demonstrated that radiotherapy (RT) has the power to trigger immunological responses that can influence disease outcomes (1–3). By induction of immunogenic cell death (ICD) and the release of tumor-associated antigens and immune adjuvants, RT can trigger pro-inflammatory reactions, promote immune cells recruitment, and break the balance of tumor immune tolerance (4). Conversely, RT can also trigger immunosuppressive signals, which can lead to tumor radioresistance (5). Treatment outcomes will ultimately depend on the net effect of pro-immunogenic and anti-immunogenic signals. Understanding the effects of radiation on the multifactorial elements of the tumor microenvironment (TME) is becoming a subject of great interest (6–8). Recent studies have shown a correlation between cancer-associated fibroblasts (CAFs), a major component of the tumor stroma (9–11), and increased radiotherapy resistance in colorectal cancer (12) and non-small cell lung cancer (NSCLC) (13). Other studies have suggested a loss of pro-tumorigenic functions in CAFs after radiation (14). Besides, it is well established that CAFs play an important role in suppressing anti-tumor immune responses in the TME, with ability to negatively affect activation, trafficking, and state of differentiation of a vast population of immune cells (15–17). However, little is known about how RT is affecting the crosstalk between CAFs and immune cells.

In the context of immune responses triggered by RT, antigen-presenting cells, in particular, dendritic cells (DCs) (18), and the induction of immunogenic cell death (ICD) are key components for effective anti-tumor response (4, 19). DCs are professional antigen-presenting cells bridging innate and adaptive immunity and are broadly divided into two major phenotypes, immature and mature DCs (20). Immature DCs are defined by a high capacity for antigen uptake and processing, with MHC-II molecules sequestered in lysosomes and low levels of antigen presentation and T cell stimulation. In contrast, mature DCs have poor endocytic capacity, with peptide-MHC complexes localized at the cell surface, securing excellent T cell priming capacity (21). Exposure of tumor lesions to ionizing radiation (IR) provokes DNA damages and may trigger ICD. ICD is characterized by the generation of damage‐associated molecular patterns (DAMPs) including extracellular exposure of calreticulin, and release of alarmins, such as high mobility group box 1 (HMGB1) and ATP (22–24). The presence of DAMPs engages receptors and ligands on dendritic cells, accelerates the engulfment of tumor-derived antigens, promotes the processing of phagocytic cargo, and activates immature DCs transition to a mature phenotype. Consequently, *via* antigen presentation, DCs stimulate specific T cell responses resulting in a robust adaptive anti-tumor immune response (20, 25). In the TME, both tumor and stromal cells can modulate infiltration, maturation, and function of DCs (18). In particular, some studies have indicated that CAFs may induce a tolerogenic phenotype on DCs. In a transplantable model of lung carcinoma, for instance, CAF-secreted tryptophan 2,3-dioxygenase (TDO2) was shown to inhibit DC differentiation and function, whereas inhibition of TDO2 improved DC function and T cell responses with decreased experimental metastasis (26). In hepatocellular carcinoma, IL-6 produced by CAFs induced a tolerogenic phenotype on DCs with decreased expression of co-stimulatory molecules and antigen-presenting receptors (CD1a, HLA-DR, CD80, CD86), and increased expression of immunosuppressive cytokines such as IL-10 and TGF-β. These CAF-educated DCs promoted tumor infiltration of immunosuppressive Tregs (CD4^+^CD25^+^Foxp3^+^) cells and decreased production of IFN-γ from CD8^+^ T cells (27). Interplay between CAFs and DCs has also been shown to affect the ability of DCs to induce the differentiation of T cells into a Th2 phenotype in pancreatic cancer, *via* CAF secretion of thymic stromal lymphopoietin (TSLP) (28).

In the RT context, CAFs are known to be highly radioresistant and may survive even ablative doses of ionizing radiation (1x18 Gy), largely reflecting their restricted tendency to proliferate, their capacity to mount solid cytoprotective responses to radiation and their high apoptotic threshold (29). In culture conditions, exposure to medium or high doses of IR does not trigger ICD in CAFs (30). However, single-high radiation doses provoke permanent DNA damage responses and the induction of premature senescence accompanied by functional changes including decreased proliferation, migration, and invasion rates (29). Radiation-induced changes have also been observed on CAF-mediated paracrine signaling. Conditioned medium (CM) from irradiated CAFs reduces the migratory capacity of endothelial cells and inhibits angiogenesis (29, 31). CAF-CM also inhibits pro-inflammatory features in M1-macrophages, and these effects are unchanged after exposing CAFs to single-high dose or fractionated-medium dose irradiation (32). In this context, levels of key CAF-secreted immunosuppressive factors such as interleukin (IL)-6, prostaglandin E_2_ (PGE2), IL-10, and transforming growth factor-beta (TGF-β), remained unchanged after radiation exposure (30–32). To better understand and exploit the immunoregulatory power of RT, it is essential to unveil how radiation modifies CAF-mediated immunoregulatory proprieties towards immune cells. In this study, we explore if CAF-mediated immunoregulatory effects on monocyte-derived DCs are changed after exposure to different radiation schemes.

## Materials and Methods

### Human Material, CAF Isolation, and Cultures

Human lung CAFs were prepared from freshly resected NSCLC tumor tissue from patients undergoing surgery at the University Hospital of Northern Norway (UNN), Tromsø, as previously described (29). Lung tumor specimens from four different patients (**Table 1**) and blood (i.e. buffy-coats) from ten unrelated healthy donors, all collected under patient written informed consent, were included in this study. All methods involving human material were performed following proper ethical guidelines and regulations under the approval of the Regional Ethical Committee of Northern Norway (REK Nord 2014/401; 2016/714; 2016/2307). NSCLC-derived CAFs were isolated by enzymatic digestion of tissues and the outgrowth method and phenotypically characterized by the presence of specific markers smooth muscle α-actin (α-SMA) and fibroblast activation protein 1 (FAP1), as described previously (29). Isolated CAFs were cultivated in Dulbecco’s modified Eagle’s medium (DMEM) (Sigma-Aldrich, St Louis, MO, USA) supplemented with 10% fetal bovine serum (FBS) (Biochrom, Berlin, Germany) and used for experimentation after the third and fourth passage (3-4 week-old cultures). Human lung cancer cell line A549 (human lung adenocarcinoma) were purchased from LGC Standards AB (Borås, Sweden). Cells were cultivated in RPMI-1640 supplemented with 10% FBS and penicillin (1%) and streptomycin (1%) in a humidified incubator at 37°C, containing 5% CO_2_ and 20% O_2_.

**Table 1:** Clinical and patient records corresponding to CAF donors used in this study.

Donors	Sex	Tumor type	T-size (mm)	Stage
1	Male	Squamous cell carcinoma	35	pT2aN0Mx
2	Male	Squamous cell carcinoma	22	pT1cN0Mx
3	Female	Adenocarcinoma	25	pT1cN0Mx
4	Male	Squamous cell carcinoma	30	pT2bN2Mx

### Irradiation of Cells

Adherent CAFs cultured in DMEM (with 10% FBS) or A549 cultured in RPMI (with 10% FBS) and grown in T-175 flasks or 24 well culture plates were irradiated when 70–90% confluent with high-energy photons producing by a clinical Varian linear accelerator and delivered in two different radiation regimens, as single-high dose (1×18 Gy) or in fractionated schemes (3×6 Gy for CAFs and 3x8 Gy for A549 cells) at 24h intervals, as previously described (29). Standard parameters for dose delivery were depth 30 mm, beam quality 15 MV, dose-rate of 6 Gy/min, and field sizes of 20×20 cm.

### Preparation of Conditioned Media

CAFs at early passages and A549 were seeded (separately) at a density of 4×10^5^ cells in T-75 tissue culture flask and incubated for 24h in DMEM and RPMI (with 10% FBS), respectively. After cell attachment and spreading, cultures were gently washed with PBS (37°C) and 6 mL of new incubation medium was added, followed by irradiation of dishes, as previously described (29). Media from CAFs and A549 cells exposed to IR (3×6 Gy and 3×8 Gy, respectively) were conditioned for 48h, after the last radiation dose. For the group exposed to 1×18 Gy, CM was conditioned between day 3 and day 5 after irradiation. Supernatants were spun down by centrifugation (2000×g, 4°C, 10 min) and then filtrated (Ø = 0.45 µm) for elimination of potential cell debris. The resulting samples were either used immediately or frozen at −80°C for later use.

### Isolation of Peripheral Blood Mononuclear Cells and Generation of Monocyte-Derived Dendritic Cells

Peripheral blood mononuclear cells (PBMCs) were isolated from human blood (i.e., buffy-coats) using Lymphoprep-TM (StemCell Technologies, Vancouver, BC, Canada) gradient centrifugation. CD14+ monocytes were isolated from the PBMCs pool using magnetic CD14^+^ Microbeads (Cat. no. 130-050-201; Miltenyi Biotec, Bergisch Gladbach, Germany). Monocytes (CD14^+^) purity and recovery were determined by CD14 antibody labeling (Cat. no. 130-113-708; Miltenyi Biotec), and cell viability by propidium iodide (PI) staining. Cells were analyzed by flow cytometry on a BD FACSAria III (BD Biosciences, San Jose, CA, USA). For the generation of immature DCs (iDCs), CD14+ monocytes were cultured in R10 medium (RPMI 1640 with 10% FBS, 1% streptomycin/penicillin, and 100 mM Sodium Pyruvate) supplemented with IL-4 (100 ng/mL; cat. no. 300-25; PrepoTech, Rocky Hill, NJ, USA) and GM-CSF (100 ng/mL; cat. no. 300-03; Prepotech) and kept in a humidified atmosphere (5% CO2, 37°C), for 5 days. The incubation medium was replaced after three days with new (pre-warmed) R10 medium supplemented with GM-CSF and IL-4. For maturation of DCs, iDCs were transferred to 6-well tissue culture plate and incubated (37°C, 48h.) in 5% CO2 humified atmosphere in the presence of the following cytokines (PrepoTech): IL-6 (15 ng/mL), IL-1β (10 ng/mL) TNF-α (50 ng/mL), and PGE2 (1 µg/mL). Absolute cell count of mature-DCs (mDCs) was determined by flow cytometry *via* light scatter signals and PI fluorescence.

### Co-Cultures and Dendritic Cell Stimulation With CAF-Conditioned Medium

In co-culture experiments, control and irradiated CAFs were established in 24-well plates (2×10^5^ cells per well). Monocytes or iDCs were thereafter added at a density of 4×10^5^ live cells per well (ratio; 2:1). Cultures with mixed cell types were further incubated for 48h at 37°C in R10 medium. Parallel procedures were implemented for experiments with CAF-CMs, but instead of cells, CAFs culture supernatants were collected, diluted (1:1) with fresh pre-warmed R10 medium, and added to the DC cultures. For differentiation studies, monocytes were exposed to GM-CSF and IL-4 immediately after initiation of co-cultures or incubations with CAF-CM. For maturation studies, iDCs were exposed to cytokine-maturation cocktail immediately after initiation of co-cultures or incubations with CAF-CM. Following treatments, DCs and supernatants were collected and used for further analysis.

### Quantitative Cell Surface Markers Expression by Flow Cytometry

DCs surface markers were analyzed by flow cytometry on BD FACSAria III using the FlowJo software, Ver.7.2.4 (Tree Star, Ashland, OR, USA). Briefly, DC preparations (3×10^5^ cells/condition) were labeled with panels of specific antibodies for each phenotype (Miltenyi Biotec). Maturation markers consisted of CD40, CD80, CD86, and HLA-DR (Cat. no. 130-099-385, 130-110-371, 130-113-571, and 130-111-943, respectively) whereas differentiation markers were identified by CD209 (DC-SIGN), CD1a, and CD14 (Cat. No. 130-101-239, 130-097-905, and, 130-113-708, respectively). Isotype controls consisted of REA control and IgG2a (Cat. no. 130-113-450 and 130-104-612, respectively). Data were obtained by flow cytometry using the following gating strategy: a) Differentiation markers: cells gated according to their scatter properties (FSC-A *vs* SSC-A), doublets exclusion (SSC-H *vs* SSC-W), and analyzed by the percentage of total cells expressing CD14, CD1a, and CD209; and b) Maturation markers: after cells were gated by scattering properties (described above), CD14^+^cells were excluded by the inverted gate and then plotted for CD1a *versus* CD209 expression. Mean fluorescence intensity (MFI) of activation markers (CD40, CD80, CD86, and HLA-DR) were analyzed in the population gated for CD1a^med/hi^/CD209^med/hi^ cells.

### Dendritic Cell Antigen Uptake

To assess DCs endocytic capacity, iDCs or mDCs (1×10⁵), previously co-cultured with irradiated or non-irradiated CAFs or CAF-CM (as described above), were incubated with FITC-labeled dextran (1 mg/mL, Cat. no. FD40S; Sigma-Aldrich) in R10 medium prepared with RPMI without phenol red (ThermoFisher Scientific, Waltham, MA, USA) for 60 min at 37°C. Non-specific binding of FITC-dextran to the cell surface was checked by keeping a control sample on ice for 60 min. Then, all samples were washed twice (centrifugation at 300×g, 5 min, 4°C) with ice-cold PBS supplemented with 0.5% of BSA and ultimately resuspended in the same ice-cold buffer. The uptake of FITC-dextran was determined by measuring MFI of the probe in cells by flow cytometry. Dead cells were excluded from the analysis by PI fluorescence. For analyses, the specific uptake of FITC-dextran was calculated by subtracting MFI of the control sample (incubated on ice) from MFI of samples incubated at 37°C.

### Dendritic Cell Migration

CCR7-dependent chemotactic responses of mDCs towards CCL19 was measured by a Boyden chamber assay. Briefly, iDCs or mDCs, previously exposed to control or irradiated CAFs/CAF-CMs (as described above), were resuspended in 200 μL of RPMI 1640 with 10% FBS at a density of 5×10⁵ cells/mL and placed in the upper compartment of a 24-well Transwell Plates (Corning; pore size 5 μm). Bottom chambers were filled with fresh pre-warmed standard fibroblast growth medium in the presence or absence of the chemoattractant CCL19 (50 ng/mL) (Cat. # 130-105-744, Miltenyi Biotec). In experiments with CAF-CM, bottom chambers were filled with CM from irradiated and control CAF cultures diluted (1:1) with fresh pre-warmed growth medium. After incubation in a humidified atmosphere (5% CO2, 37°C, 3h), cells that had migrated into the lower compartment were harvested and counted in a hemocytometer under light microscopy.

### Assessment of T Cell Priming Capacity of mDCs

Purified allogeneic naive CD4^+^ T cells were isolated from PBMCs-pool using magnetic Naive CD4^+^ T Cell Isolation Kit II (Cat. no. 130-094-131; Miltenyi Biotec) and labeled with carboxyfluorescein succinimidyl ester (CFSE) (Cayman Chemical, Ann Arbor, MI, USA) for 15 min at 37°C (1:400 dilution in PBS). The purity of isolated enriched naïve CD4^+^ T cells was determined by CD4, CD45RO, and CD45RA antibody labeling (cat. no. 130-113-776, 130-109-507, and 130-098-187; Miltenyi Biotec). CSFE-stained CD4^+^ T cells (5×10⁵ cells/mL) were co-cultured with iDCs or mDCs (1×10⁶ cells/mL, ratio 1:2), previously incubated with irradiated or control CAFs/CAF-CM for 48h (as described above), in MLR medium (RPMI 1640, 2 mM L-glutamine, non-essential amino acids, 0.1 mM sodium pyruvate, 5% AB serum) for 7 days at 37°C, 5% CO₂. Proliferation of CD4^+^ T cells was determined by measuring CFSE fluorescence intensity by flow cytometry. Cell debris and dead cells were excluded from the analysis by scatter signals and PI fluorescence.

### Quantitative Cytokine Release by ELISA

Quantitative determinations of IL-10 and IL-12 in supernatants (diluted 1:10) from co-cultures (DCs/CAFs) or DCs cultures stimulated with CAF-CM were determined using ELISA kits (R&D Systems, Minneapolis, MN, USA) according to the manufacturer’s instructions. For TSLP quantification, CAFs were cultured at T-75 tissue culture flasks in DMEM (with 10% FBS) and exposed to fractionated medium-dose of IR (3×6 Gy) or stimulated with 10 ng/mL of TNF-α (PrepoTech) to induce TSLP secretion by CAFs (28). After 48h, CM were collected, spun down by centrifugation (2000x g, 4°C, 10 min), filtrated (Ø = 0.45 µm) and stored at -80 °C. Samples (diluted 1:2) were analyzed using Human TSLP ELISA Kit (Abcam, Cambridge, UK). Absorbance at 450 nm for each sample was analyzed by SpectraMax Plus 384 Microplate Reader (Molecular Devices, CA, USA).

### Immunoblotting

Whole-cell extracts from DCs or CAFs were prepared in RIPA buffer (Cell Signaling, Boston, MA, USA) plus Complete Protease and Phosphatase Inhibitor Cocktail (ThermoFisher, cat.no. 78440). Total cell-associated proteins were separated on 10% SDS-polyacrylamide gel electrophoresis (PAGE) and transferred onto a PVDF membrane. The membrane was blocked with 1% BSA in tris buffered saline, 0.1% Tween 20 (TBS-T) for 2h at room temperature, and then incubated (overnight, 4°C) with primary antibodies (anti-GAPGH, cat. no. 5174; anti-STAT3, cat. no. 4904; anti-p-STAT3 (S727), cat.no. 34911; anti-p-STAT3 (Y705), cat-no. 9145; anti-NF-κB/p65, cat.no. 8242; anti-p-NF-κB/p65, cat.no 3033; Cell Signaling; anti-TDO2, cat.no. ab76859) diluted 1:1000 (in TBS-T with 1% BSA). Subsequently, the membrane was washed 5x in TBS-T and then incubated with an anti-rabbit or anti-mouse HRP-conjugated secondary antibody (diluted 1:2000; Cell Signaling) for 1h at room temperature. Finally, proteins transferred to the membrane were visualized with Enhanced Chemiluminescence at ImageQuant LAS 4000 CCD (GE Healthcare Bio-Sciences, PA, USA). Relative intensity was assessed using ImageJ software.

### Statistical Analysis

All statistical analyses were performed using GraphPad Prism (GraphPad Software, Inc, La Jolla, CA). Comparison of data between experimental groups was analyzed using the Brown-Forsythe and Welch ANOVA test, and significance values were adjusted by Dunnett’s T3 correction for multiple comparisons. Outcomes of Western blot experiments were analyzed using the 2way ANOVA test, and significance values were adjusted by Dunnett correction for multiple comparisons. The level of significance was set at *p* < 0.05. Results were presented in graphs, where each donor was plotted as an individual dot in the dataset. In ELISAs, only readings above the detection limit of the assay are shown.

## Results

### CAF-Mediated Effects on DC Phenotypic Differentiation and Maturation

**Figure 1:**
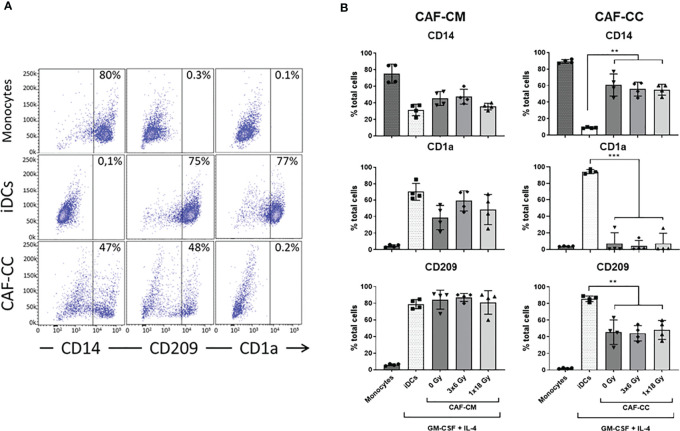
Effects of CAFs on DC differentiation markers. Monocytes stimulated with GM-CSF and IL-4 were incubated for 6 days with conditioned medium from irradiated or non-irradiated CAFs (CAF-CM, left panels) or in co-cultures (CAF-CC, right panels). Resulting expression of iDC cell surface markers CD14, CD1a, and CD209 were evaluated by flow cytometry. **(A)** Representative dot plots of the percentage of expression of CD14, CD209, and CD1a in monocytes, iDCs, and monocytes stimulated with GM-CSF and IL-4 in co-culture with CAFs. **(B)** Bar graphs represent mean ( ± SD) values from flow cytometry analysis of 4 different CAF donors, measured independently. Pattern columns indicate surface levels in control monocytes and iDC cultures. Results are expressed as percentage of total cells. Brown-Forsythe and Welch ANOVA test and *p-values* were determined between iDCs and non-irradiated CAFs, iDCs, and the two irradiated CAF-groups separately. **p ≤ 0.01

To investigate the effects of IR on CAF-mediated regulation of monocyte-to-DC trans-differentiation, peripheral blood monocytes from health donors (CD14^+^ cells – 89% purity) were cultured in medium containing DC differentiation cytokines (IL-4 and GM-CSF) in the absence or presence of conditioned medium from irradiated or non-irradiated CAFs (CAF-CM) or alternatively in (CAF-DC) co-cultures (CAF-CC). DCs were not differentiated from peripheral blood monocytes of cancer patients mainly because of an individual constitutional characteristic of the patients that reflects on phenotypic and functional alterations in mo-DC (33–35). Following incubation for 6 days, non-adherent cells were harvested and phenotyped by flow cytometry. Considering the potentially different effects triggered by different radiation schemes, we compared the effects of fractionated and single-high dose radiation. **Figure 1** shows the percentage of cells expressing signature DC surface marker molecules CD1a and CD209 (DC-SIGN), and the lipopolysaccharide co-receptor CD14, as determined by flow cytometry. Transformed monocytes presented the typical phenotypic profile of immature DCs (iDCs) defined by CD1a^high^, CD209^high^, and CD14^medium/low^ (**Figure 1A**). As shown in **Figure 1B**, the presence of CAFs clearly interferes with monocyte-DC differentiation, especially in co-culture conditions. DCs in co-culture with CAFs expressed significantly lower levels of CD1a (*p* ≤ 0.001) and CD209 (*p* ≤ 0.01) and increased expression of CD14 (*p* ≤ 0.01). In experiments with CAF-CM, only the expression of CD1a was slightly decreased when compared to iDCs controls. Of note, no statistically significant differences were observed in the expression of any of the receptors when comparing irradiated with non-irradiated CAF conditions, both in CC- or CM-conditions.

**Figure 2:**
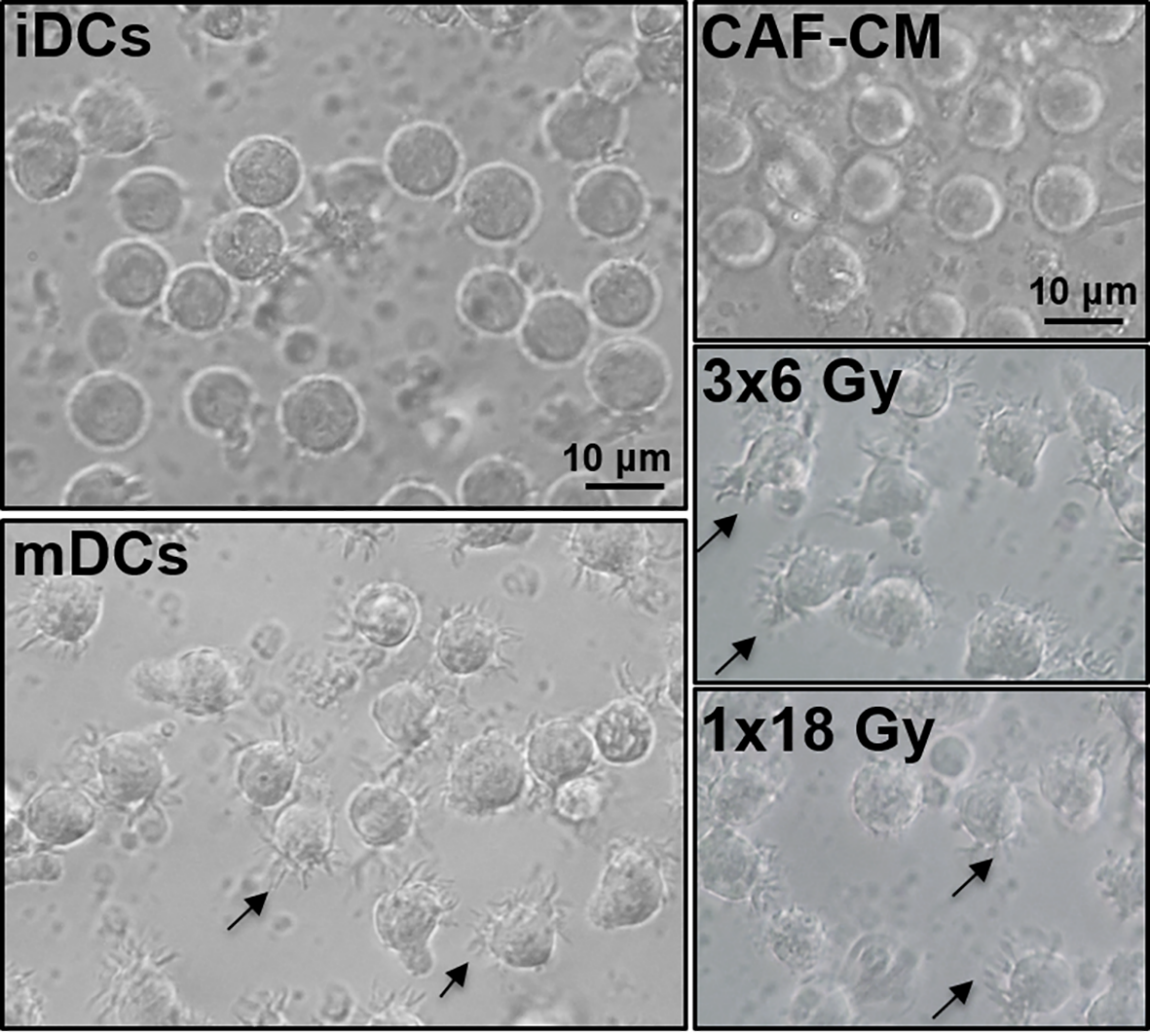
Effects of CAF-CM on DC phenotype. Gross morphology of DC cultures by phase-contrast microscopy. Immature DCs show a classical round morphology whereas mature DCs present characteristic dendrites. Incubations with CAF-CM partially revert the phenotype of mDCs into iDCs (top-right panel), whereas this effect is lost in the conditioned medium from CAFs exposed to 3x6 Gy. Cells presenting dendrites in control mDCs and fractionated radiation (3x6 Gy) CAF-CM groups are shown by arrowheads. Scale Bars = 10 μm.

To induce DC maturation, iDCs were exposed for 2 days to a maturation-cocktail of cytokines comprising IL-6, IL-1β, TNF-α, and PGE2. Matured DCs (mDCs) cultured in the absence or presence of CM from irradiated or non-irradiated CAFs revealed differences in their morphology (**Figure 2**). Whereas conventional mDC presented abundant cellular protrusions and membrane ruffling, DCs maturated in the presence of CAF-CM appeared with a typical iDC morphology; large rounded cells with eccentrical nucleus location and few cellular protrusions. However, this effect was to some extent abolished in cells cultured in the presence of irradiated CAF-CM.

**Figure 3:**
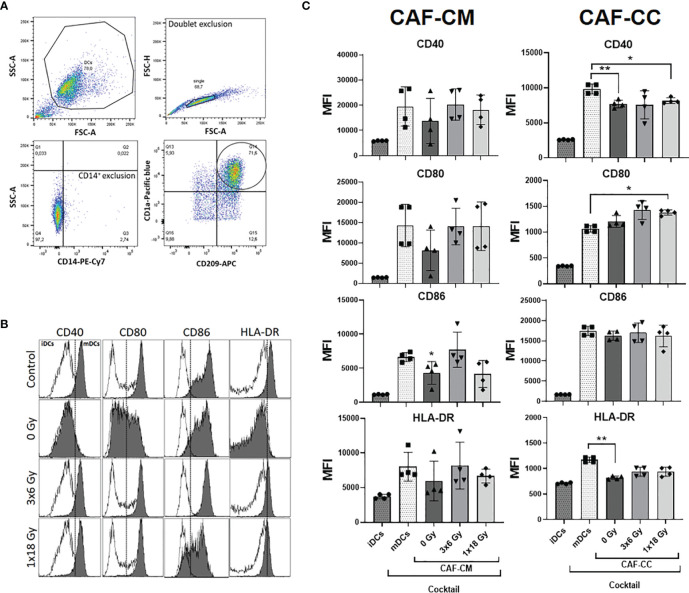
Effects of CAFs on DC activation markers. Immature DC (iDCs) stimulated with a maturation cytokine cocktail were incubated for 48h with conditioned medium from irradiated or non-irradiated CAFs (CAF-CM, left panels) or in co-cultures (CAF-CC, right panels). Resulting expression of mDC cell surface markers CD40, CD80, CD86, and HLA-DR was evaluated by flow cytometry. **(A)** Gating strategy used to analyze the expression of activation markers in DCs. **(B)** Representative histograms of expression by mean fluorescence intensity (MFI) of CD40, CD80, CD86, and HLA-DR in DCs stimulated with maturation cocktail in culture with conditioned medium from non-irradiated and irradiated CAFs (1 donor). **(C)** Bar graphs represent mean ( ± SD) values from flow cytometry analysis of four-4 different CAF donors, measured independently. Pattern columns indicate protein surface levels in control iDC and mDC cultures. Results are expressed as percentage of total cells. Data represent mean ( ± SD) values from 4 different CAF donors measured independently. Brown-Forsythe and Welch ANOVA test and *p-values* were determined between control and non-irradiated CAFs, mDCs, and the two irradiated CAF-groups individually. *p ≤ 0.05, **p ≤ 0.01.

Next, we determined whether CAFs could affect DC maturation. Surface expression of antigen-presenting receptor HLA-DR and the co-stimulatory receptors CD40, CD80, and CD86 were analyzed following the gating strategy described in **Figure 3A**. As expected, stimulation of iDC with the cytokine maturation cocktail enhanced the expression of CD40, CD80, CD86, and HLA-DR as shown in **Figure 3B**. However, cells incubated with CAF-CM showed decreased expression of CD80 (*p* = 0.4256), CD86 (*p* ≤ 0.05), and HLA-DR (*p* = 0.8375), with same tendency also for CD40. Similarly, in co-culture conditions, CAFs were exerting inhibitory effects on surface expression of CD40 (*p* ≤ 0.01) and HLA-DR (*p* ≤ 0.01), compared to mDCs controls. No statistical differences were observed when comparing DC phenotype cultured with both irradiated and non-irradiated CAF-CM or CAF-CC. Nevertheless, CAFs irradiated with fractionated medium-doses showed a tendency to reverse the paracrine effect on the expression of DC surface receptors exerted by control CAFs (**Figure 3C**).

### Effects of CAFs on DC Cytokine Release

**Figure 4:**
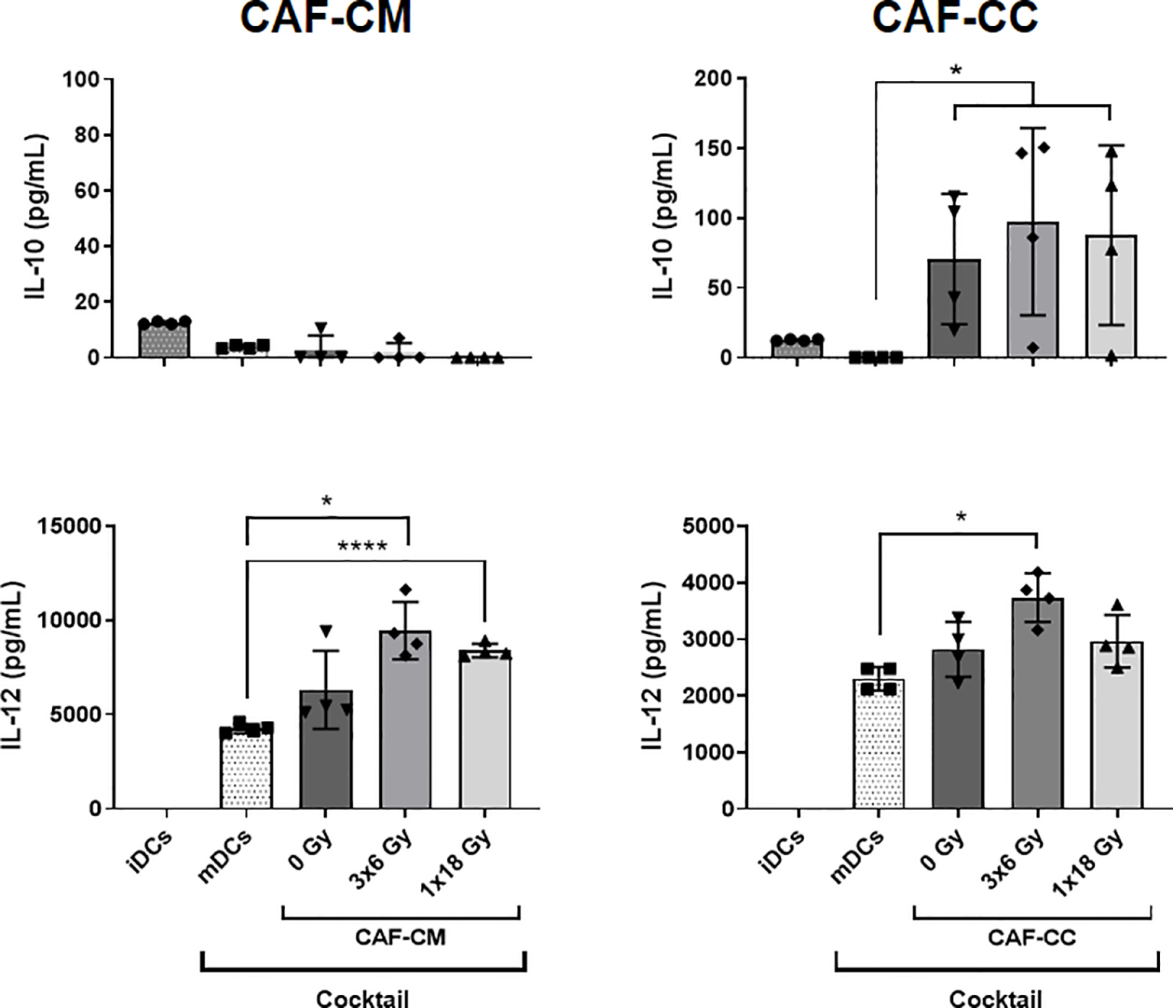
CAF-induced cytokine secretion by DCs. Monocyte-derived dendritic cells in non-stimulated or stimulated conditions were incubated for 48h with irradiated or non-irradiated CAF conditioned medium (CAF-CM, left panels) or in co-cultures (CAF-CC, right panels). Resulting levels of IL-10 (top panels) and IL-12 (lower panels) found in supernatants were quantified by ELISA assays. Data represent the mean ( ± SD) values from 4 different CAF donors measured in duplicates. Brown-Forsythe and Welch ANOVA test and-values were determined between mDCs and non-irradiated CAF-CM, mDCs *vs* irradiated CAFs. *p ≤ 0.05, ****p ≤ 0.0001.

To explore further the immunoregulatory properties exerted by CAFs on DCs, we quantified protein levels of IL-10 and IL-12 in culture supernatants from DCs exposed to CAF-CM or in co-cultures under the stimulus of maturation cocktail. Of note, CAF exposed to DCs maturation cocktail did not secrete significant levels of IL-10 and IL-12 as shown in **Supplementary Figure 3**. Results from corresponding analyses of IL-10 and IL-12 are presented in **Figure 4**. The amount of IL-10 was very low in supernatants of iDCs and nearly undetectable in mDCs, treated or not with CAF-CM. However, levels of IL-10 were considerably increased (*p* ≤ 0.05) in CAF-CC experiments, including both irradiated and non-irradiated CAFs. On the other hand, levels of IL-12 were undetectable in iDCs cultures but considerably increased in mDCs supernatants. Interestingly, a significant increase in secreted levels of IL-12 was observed in mDCs cultures in the presence of CM from the two irradiated-CAF groups (3x6 Gy, *p* ≤ 0.05; 1x18 Gy, *p* ≤ 0.0001). Moreover, increased IL-12 secretion was also observed when mDCs were co-cultured with CAFs irradiated at 3x6 Gy (*p* ≤ 0.05), whereas minor differences were seen between mDC alone or co-cultured with non-irradiated or 1x18 Gy irradiated CAFs (**Figure 4**).

### CAF-Mediated Effects on DC Functions

**Figure 5:**
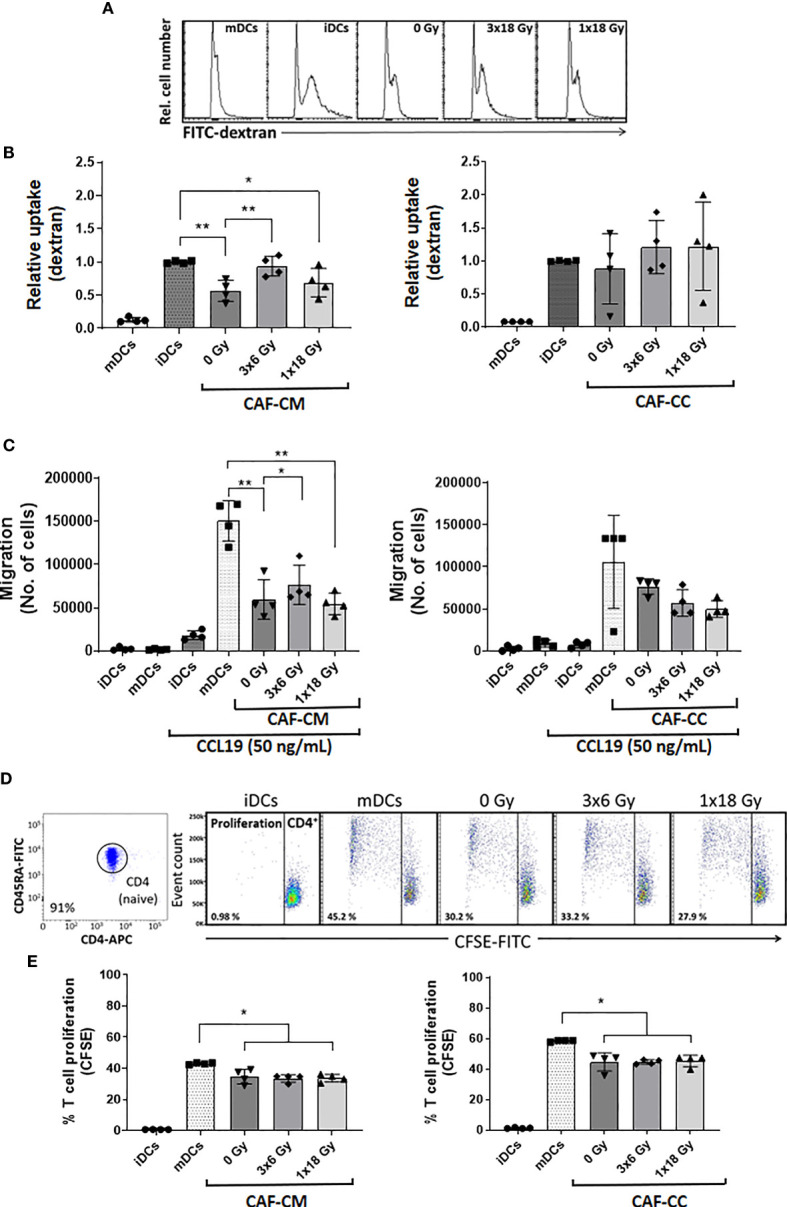
CAF-mediated effects on DC functions. Antigen uptake capacity by DCs was analyzed by flow cytometry. After initial treatments, DCs were cultured for 60 min in the presence of FITC-dextran. Relative FITC-dextran uptake was calculated by subtracting MFI of cells incubated for 60 min on ice from MFI of cells allowed to internalize antigen during 60 min at 37°C. **(A)** Representative histograms indicating MFI of FITC-dextran uptake by mDCs. **(B)** Bar graphs represent mean ( ± SD) values from flow cytometry analysis of four-4 different CAF donors measured independently. **(C)** DC migration rates were measured by the Boyden chamber assay. The total number of cells that migrated towards a CCL19 gradient during 3h was determined for each experimental group. **(D, E)** DCs T cell priming capacity was analyzed by CFSE-dilution assay. Naive CD4^+^ T cells were co-cultured with mDCs (ratio 2:1) in the presence of CAFs or CAF-CM for 7 days and the percentage of proliferating T cells was determined by flow cytometry. **(D)** Representative dot plots indicating the percentage of purity of CD4RA naive T cells and the percentage of the proliferation of CD4 cells co-cultured with mDCs. **(E)** The bar graphs represent mean ( ± SD) values from flow cytometry analysis of 4 different CAF donors measured independently. Dead cells were excluded from the analysis by PI fluorescence. Brown-Forsythe and Welch ANOVA test and *p-values* were determined between controls and non-irradiated CAFs, mDCs, and the two irradiated CAF-groups individually. *p ≤ 0.05, **p ≤ 0.01.

We sought to explore the capacity of CAFs to modulate key functional properties on DCs. First, we analyzed changes in the endocytic capacity of DCs by exposing DC cultures for 1h to soluble FITC-dextran by flow cytometry. iDCs had the highest antigen uptake capacity, reflected in an increased MFI, as compared to mDCs (**Figure 5A**). Of note, immature DCs exposed to CM from non-irradiated (*p* ≤ 0.01) and high-dose irradiated (*p* ≤ 0.05) CAFs presented a significant decrease in uptake capacity, as compared to untreated iDCs. Notably, DC uptake of FITC-Dextran in the (3x6 Gy) irradiated CAF-CM group was comparable to (high-uptake) iDCs controls. However, in CAF-CC, no significant differences were observed between iDCs control and CAF-treated groups (**Figure 5B** and **Supplementary Figure 4**).

Second, we assessed the capacity of CAFs to modulate the migratory capacity of mDCs. DC migration depends on the surface expression of C-C Motif Chemokine Receptor 7 (CCR7) (36). CCR7 expression increase during DC maturation, acting as a receptor for the constitutively expressed chemo-attractants CCL21 and CCL19 (37). As illustrated in **Figure 5C**, mDCs display much higher migration rates as compared to iDCs. Matured DCs exposed to any of the three different CAF-CMs resulted in decreased migratory capacity with significant values from both control CAF-CM (*p* ≤ 0.01) and irradiated CAF-CM (3x6 Gy, *p* ≤ 0.05; 1x18 Gy, *p* ≤ 0.01), although DCs exposed to (3x6 Gy) irradiated CAF-CM displayed a significantly increased migration rates compared to other CAF-CM groups. In CAF-CC conditions, no significant differences in migration were observed between mDCs control and CAF-treated groups (**Figure 5C**).

Third, we tested how CAFs may influence the mDC capacity to induce T cell proliferation in a mixed lymphocyte reaction (MLR). To this end, we isolated and labeled allogeneic naive CD45RA^+^/CD4^+^ T cells (purity of 91%, **Figure 5D**) from whole blood with CFSE. The resulting fluorescent T cells were incubated with mDCs (2:1 ratio) that were previously conditioned with CAF-CM or CAF-CC for 48h. T cell proliferation rates were measured by CFSE dilution assay and analyzed by flow cytometry (**Figure 5D**). mDCs were able to stimulate T cells proliferation much more efficiently than iDCs. However, a significant decrease in T cell proliferation was observed when mDCs were pre-exposed to irradiated or non-irradiated CAF-CM (*p* ≤ 0.05), or with CAF-CC (*p* ≤ 0.05) (**Figure 5E**). In both conditions, ionizing radiation, applied in single or fractionated doses, did not change the CAF-mediated suppressive effects on DC-mediated T cell proliferation.

### Alterations on STAT3 Signaling and NF-κB/p65 Activation in CAF-Exposed DCs

**Figure 6:**
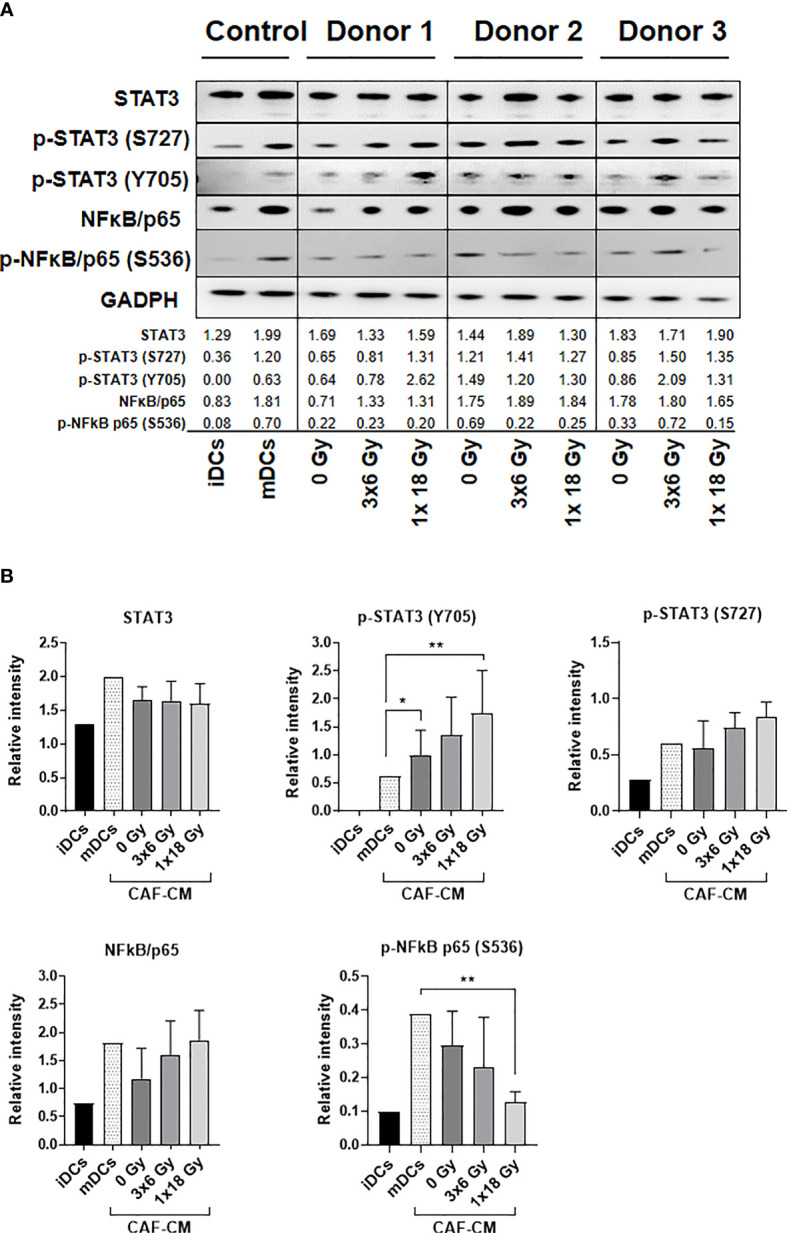
Alterations on STAT3 signaling and NF-κB activation in DCs exposed to CAF-CM. **(A)** Western blot analysis, using anti-STAT3, p-STAT3 (S727), p-STAT3 (Y705), NF-κB/p65, and p-NF-κB/p65 (S536) on whole DC cell lysates stimulated with irradiated and non-irradiated CAF-CM. Results were normalized against GAPDH expression and the results of phosphorylated proteins were normalized against the respective total proteins. In **(B)**, the relative intensity of the bands corresponding to **(A)**, determined by densitometry, is shown as a bar graph. Data represent mean (± SD) values from 3 different CAF donors. Two-way ANOVA test and *p-values* were determined between non-irradiated CAFs, mDCs, and the two irradiated CAF-groups individually. *p ≤ 0.05, **p ≤ 0.01.

To investigate CAF-mediated DC alterations in NF-κB/p65 and STAT3 signaling pathways, DCs were exposed to irradiated and non-irradiated CAF-CM (from 3 different donors) during DC maturation, and expression of total and phosphorylated NF-κB/p65 and STAT3 were analyzed by immunoblotting (**Figure 6A**). STAT3 signaling analysis showed that phosphorylation of STAT3 at Y705 was enhanced in DCs incubated with CAF-CM, and significantly increased in cells exposed to CM from irradiated groups as compared to non-irradiated (3x6 Gy, *p* ≤ 0.05), but even more pronounced when compared to the group irradiated with a single high-dose (*p* ≤ 0.001). No significant differences between irradiated and non-irradiated groups were observed on STAT3 phosphorylation at S727, although irradiated-CAF-CM showed a tendency to increase the phosphorylation at S727 (**Figure 6B**). Similar patterns were observed in the activation of NF-κB/p65; expression of total NF-κB/p65 were increased in DCs exposed to irradiated-CAF CM. However, phosphorylation at S536 was lower in DCs exposed to CM from CAFs irradiated with high-single dose (*p* ≤ 0.001). Of note, CM from non-irradiated CAFs attenuated both total and phosphorylated NF-κB/p65 in DCs (**Figure 6B**). In summary, these data indicate that: 1) secreted factors from CAFs can interfere with cytokine-induced NF-κB/p65 and STAT3 signaling on DCs, and 2) ionizing radiation abrogates to some degree the CAF-mediated effects.

### Effect of Radiation on CAFs Secretory Profile and Paracrine Signaling

**Figure 7:**
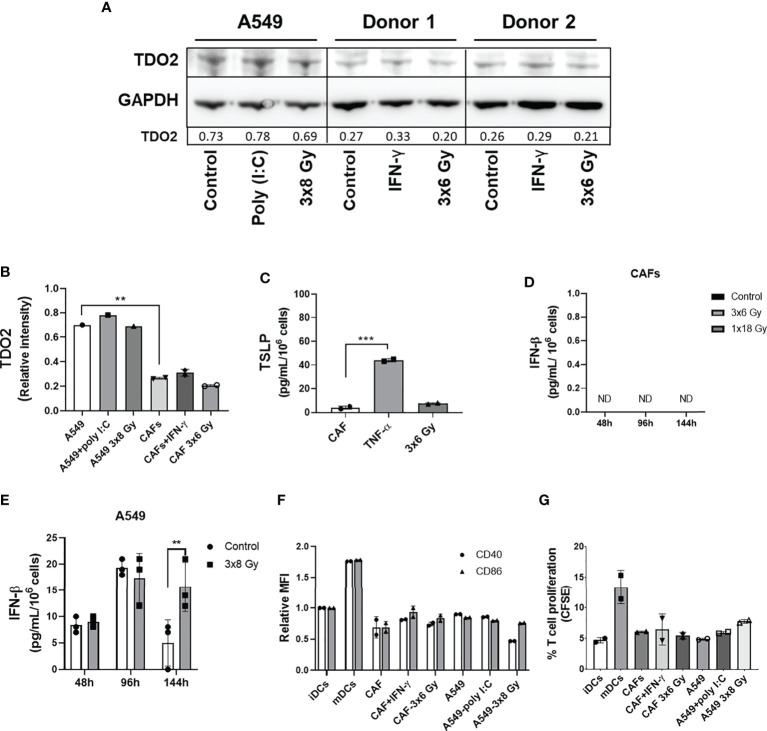
Effect of radiation on expression of CAF immunomodulators and DC immunoregulation by irradiated CAFs supernatants. Cultures were irradiated with fractionated doses of 6 Gy (CAFs) and 8 Gy (A549) during three consecutive days. **(A)** Protein expression of TDO2 was determined by Western blotting and results were normalized against GAPDH expression. For positive controls, A549 cells were stimulated with 0.1 µg/mL of poly (I:C) and CAFs were stimulated with 10 ng/mL of IFN-γ. In **(B)**, relative intensity of the bands corresponding to panel A, determined by densitometry, is shown as a bar graph. Data represent mean (± SD) values from two different CAF donors. In **(C)**, the resulting levels of TSLP were quantified by ELISA. For positive controls, CAFs were stimulated with 10 ng/mL of TNF-α. Data represent mean ( ± SD) values from two different CAF donors measured in duplicates. The effect of radiation on the secretion of IFN-β by CAFs **(D)** and by A549 cells **(E)** was analyzed by ELISA assay. IFN-γ secretion in supernatants was determined 48, 96, and 144h post-irradiation. In **(F)**, the resulting expression of co-stimulatory markers CD40 and CD86 on DCs treated with CM from non-irradiated and irradiated CAFs (3x6 Gy) and A549 tumor cells (3x8 Gy) were evaluated by flow cytometry. In **(G)**, naive CD4+ T cells were co-cultured for 7 days with DCs (ratio 2:1) stimulated with CM from non-irradiated and irradiated CAFs (3x6 Gy) and A549 tumor cells (3x8 Gy), and percentage of proliferating T cells was determined by flow cytometry. Dead cells were excluded from the analysis by based on PI fluorescence. Data represent the mean ( ± SD) values from triplicates. Two-way ANOVA test and *p-values* were determined between non-irradiated *vs* irradiated cells. Brown-Forsythe and Welch ANOVA test and *p-values* were determined between control and TNF-α stimulated CAF-CM and control *vs* irradiated CAFs. ***p ≤ 0.001, ***p ≤ 0.001. ND, not detected.

Earlier studies on the crosstalk between CAFs and DCs in different cancer models suggest that CAF-mediated immunoregulation on DCs is achieved *via* release of the cytokine thymic stromal lymphopoietin (TSLP) or expression of the catalytic enzyme tryptophan 2,3 dioxygenase (TDO2). To investigate the possible effects of fractionated medium-dose radiation on the expression of TDO2 and TSLP, both CAFs and A549 lung tumor cells were irradiated (3x6 Gy and 3x8 Gy, respectively) and the expression of TDO2 was analyzed in cell lysates by immunoblotting (**Figure 7A**), whereas TSLP release was analyzed in supernatants by ELISA (**Figure 7C**). Lung tumor cells (A549) showed significantly higher expression of TDO2 than CAFs (*p* ≤ 0.01) (**Figure 7B**). However, no significant differences were observed between irradiated and non-irradiated CAFs or A549 groups on TDO2 expression (**Figures 7A, B**). Positive controls, represented by A549 cells stimulated with poly (I:C) and CAFs stimulated with IFN-γ, showed a slight increase in TDO2 expression compared to untreated cells. Analysis by ELISA showed no differences in the secretion of TSLP between irradiated and non-irradiated CAFs whereas CAFs treated with TNF-α (positive controls) secreted significantly higher levels of TSLP as compared to the control group (*p* ≤ 0.001) (**Figure 7C**). In summary, these data indicate that the depriving effects mediated by fractionated medium-dose radiation on CAF-induced DCs tolerogenic phenotype are not dependent on the regulation of TDO2 or TSLP expression.

Further, we characterized the effect of IR on IFN-β secretion by CAFs. Analysis by ELISA showed that IFN-β levels were not detectable in the CM from non-irradiated and irradiated CAFs (3x6 Gy and 1x18 Gy) after 48, 96, and 144h of IR exposure (**Figure 7D**). On the other hand, A549 tumor cells exposed to 3x8 Gy secreted increased levels of IFN-β (*p* ≤ 0.01) 144h post-IR exposure, as compared to non-irradiated cells (**Figure 7E**). Next, we determined whether CM from fractionated irradiated CAFs (3x6 Gy) could induce DC maturation through the analysis of co-stimulatory receptors CD40 and CD86, and the induction of T cell proliferation in MLR. As shown in **Figure 7F**, no differences in the expression levels of CD40 and CD86 were observed in DC stimulated with CM from irradiated or non-irradiated CAFs (3x6 Gy) and A549 tumor cells (3x8 Gy). CAFs stimulated with IFN-γ (positive control) showed a slight increase in CD86 expression on DCs compared to cells cultured with CM from untreated CAFs (**Figure 7F**). Similar patterns were observed in the ability of DCs to stimulate T cell proliferation. We show that iDCs incubated with CM from non-irradiated and irradiated CAFs or A549 tumor cells were not able to induce CD4^+^ T cell proliferation to the extent of mature DC controls (**Figure 7G**).

## Discussion

In the present study, we have investigated how CAFs from lung tumors influence monocyte-derived DC differentiation, maturation, and functions *in vitro*; and whether ionizing radiation is able to modify the CAF-mediated immunoregulatory features on DCs. We have observed that: (i) CAFs hamper monocytes differentiation into DCs; (ii) CAFs induce a tolerogenic phenotype on mature DCs, as evidenced by decreased expression of activation markers (CD80, CD86, CD40, and HLA-DR) and reduced functional properties (migration, antigen uptake, and CD4^+^ T cell priming); (iii) IR applied in fractionated medium-doses (3x6 Gy) reverts some of the CAF-mediated effects on DCs; (iv) IR induces changes in CAF paracrine factors that modulate the activation of NF-κB/p65 and STAT3 signaling pathways on DCs; (v) neither TSLP nor TDO2 expression in CAFs is altered by radiation exposure.

Early on, we showed that cytokine-induced monocyte differentiation into DCs is hampered in the presence of CAFs. DCs exposed to both irradiated and non-irradiated CAFs showed increased levels of the monocyte marker CD14 and decreased expression of DC signature molecules CD1a and CD209 (DC-SIGN). Some authors consider residual CD209^+^ cells as macrophages based on their morphology and co-expression of CD14 (38). However, we did not confirm whether CAF-educated monocytes were macrophages or not. The failure of those cells to downregulate CD14 could be attributed, in part, to the secretion of IL-6 by CAFs. Fibroblast-derived IL-6 has previously been shown to affect differentiation of monocytes into macrophages rather than DCs, by inducing expression of functional M-CSF on monocytes (39). In a different study, Spary et al. (40) demonstrated that the high expression of IL-6 produced by stromal cells (α-SMA^+^ cells), in prostate cancer tissue, is correlated with an induction of tolerogenic DCs phenotype, characterized by cells expressing high surface levels of CD14 and PD-L1. We have previously shown that lung CAFs represent an important source of IL-6 into the TME, however, exposure to IR does not seem to modify substantially IL-6 release from CAFs in cultures (31, 32). Likewise, Kalinski et al. (41) suggested that monocyte differentiation into DCs could be regulated by PGE2, with subsequent activation of cyclic nucleotide signaling pathways on DCs, blocking both down-regulation of CD14 and up-regulation of immature DC marker CD1a. In agreement with our observations, CAFs in cultures have been shown to produce PGE2, but its expression remains stable upon irradiation (30). Additionally, TGF-β and IFNs have been identified as negative regulators of CD209 expression and, consequently, inhibit CD209-dependent binding of HIV-1 to differentiated DCs (42). In the radiation context, we have previously shown that CAF-secreted TGF-β is not changed after exposure to single high-dose or fractionated medium-dose IR (30, 32) and in this study, we show that IFN-type I is undetectable in supernatants from irradiated or control CAFs (**Figure 7**). Collectively, knowledge generated on the irradiated CAF secretory profile, showing unchanged levels of relevant immunosuppressive signals, may explain to some extent the observation that CAF-induced effects on monocyte-to-DC differentiation is not changed after IR.

Moreover, we show that iDCs in the presence of CAF-CM display reduced antigen uptake capacity, which can be correlated with the down-regulation of CD209 expression in the target cells (43). Importantly, we showed that fractionated medium-dose radiation abrogates the CAF-mediated paracrine effect on DCs antigen uptake. However, we see no differences in the expression of CD209 in DCs exposed to irradiated or non-irradiated CAF-CM, suggesting that CAFs could regulate expression of different receptors involved in the uptake of dextran, e.g. mannose receptor (CD206), langerin receptor (CD207) or scavenger receptors (44, 45). Furthermore, iDCs were maturated with a cocktail of cytokines, including TNF-α, IL-1β, IL-6, and PGE2. The rationale for the use of this cocktail is to enhance the pro-inflammatory effects and to attempt mimicry of the RT-induced inflammatory tumor microenvironment. We found that both CAF paracrine factors and cell-contact mediated mechanisms were involved in the induction of a tolerogenic phenotype in DCs, characterized by lower expression of co-stimulatory markers, enhanced IL-10 release, along with reduced antigen capture, lower migratory capacity, and T cell priming capacity. Differences in DCs phenotype and function were observed between experiments conducted with CAF conditioned medium and in co-culture conditions. We hypothesize that the presence of concentrated soluble factors on CAF conditioned medium can significantly modulate the functions of DCs compared to those in DCs co-cultured with CAFs. On the other side, in co-culture conditions, we must consider effects coming from both soluble signals and cell-cell contacts, and effects exerted in two directions, whereas in CM conditions we only observe effects exerted by CAFs on DCs mediated by soluble factors. Previous studies demonstrated that both cell-cell interaction (fibroblasts/DCs) and soluble factors secreted from fibroblasts could act as potent regulators of DC differentiation and function (46–48). Collectively, our data are in line with previous studies that have demonstrated a direct connection between tumor-associated fibroblasts and induction of tolerogenic DCs in hepatocellular carcinoma (27), lung (26), and pancreatic (28) cancers. Some CAF-released suppressive soluble mediators, like TGF-β, IL-6, or PGE2, as well as VEGF, TDO, and TSLP, have been shown to impair DC maturation, co-stimulatory molecule expression, and antigen-presenting function (17, 26, 28, 42). We have shown in previous *in vitro* studies that protein levels of IL-6, TGF-β, VEGF, or PGE2, secreted by CAFs, are not significantly modified after direct radiation exposure (31, 32). In this study, we additionally show that CAF-derived TSLP and TDO2 levels are unchanged after exposing CAFs to fractionated medium-dose IR. The results suggest that the loss of CAF-mediated effects over DC following IR is not dependent on the modulation of previously highlighted soluble mediators.

Furthermore, we observed that IR applied as fractionated medium-doses, but not as single high-doses, promotes the loss of CAF-mediated immunosuppressive effects on DCs *via* paracrine signaling. In an earlier preclinical study by Dewan et al., it was demonstrated that interferon type-I responses and abscopal effects in combination with immune checkpoint inhibitors are only accomplished when radiation is given in fractionated medium-high doses (3x8Gy) (49). Later on, the same group has shown that repeated medium-high doses, below a threshold of 10-12 Gy, do not induce DNA exonuclease Trex1, and thus elevates interferon-β production in tumor cells, which promotes recruitment and activation of Batf3-dependent DCs (50). Importantly, on the opposite of what it is normally observed with tumor cells, we do not see type-I IFN responses induced by radiation in CAFs (**Figure 7D**). Accordingly, we do not observe activation of iDC by irradiated CAF supernatants. These outcomes are consistent with the highly radioresistant nature and the remarkable cytoprotective responses displayed by CAFs in stressful scenarios and suggest that CAFs do not contribute to the release of ICD signals and immune adjuvants following radiotherapy. Altogether, these observations indicate that radiotherapy, applied in hypofractionated medium-dose schemes to tumors, has the potential to achieve both induction of immune activation (from tumor cells) and reduction of immunosuppression (from CAFs) concomitantly.

To explore possible mechanisms behind the effects of irradiated CAF-CM on DCs, we investigated the activation of NF-κB and STAT3 signaling pathways on DCs. Activation of the NF-κB pathway is a central component of DC activation (51–53) and has also been implicated in the cellular response to radiation (54). During DC maturation, through pro-inflammatory stimulus (e.g., TNF-α, IL-1, IL-6, PGE2, between others), the canonical NF-κB signaling is activated (55). This signaling cascade involves phosphorylation and degradation of the inhibitory complex IκB, with release of NF-κB heterodimer p65/p50, followed by nuclear translocation and upregulated transcription of NF-κB (51, 52). On the other hand, induction of STAT3 signaling in immature myeloid cells may prevent DCs from differentiating into mature DCs (56). Several tumor-associated factors that are known to suppress DC maturation, including IL-6, IL-10, and VEGF, are activators of STAT3 (57). In this study, we observed that CM from irradiated and non-irradiated CAFs keeps NF-κB activation at lower levels during DC differentiation. However, CM from single high-dose irradiated CAFs (1x18 Gy) promoted a decreased activation of the canonical NF-κB signaling pathway. Li et al. (58) demonstrated that DCs stimulated with sera from patients with NSCLC had systematic functional deficiencies correlated with simultaneous repression of NF-κB and STAT3 signaling pathways. In our study, a slight decrease of phosphorylated p-STAT3 (S727) was observed in cells stimulated with CAF-CM. However, CM from both irradiated CAF-groups increased expression levels of p-STAT3 (Y705) on DCs. Based on those shreds of evidence, we hypothesize that IR could modulate a pro-inflammatory CAF-secretome that regulates various downstream target genes, including cytokines, chemokines, receptors, and transcription factors that are relevant for DC functions.

Our study adds new knowledge to the important crosstalk between CAFs and dendritic cells in the irradiated tumor microenvironment. The results show that lung CAFs lower the expression of antigen-presenting molecules and co-stimulatory receptors in monocyte-derived DCs, thus inhibiting to some extent their antigen presentation capacity and their capability to activate cytotoxic T cell responses. Importantly, we have demonstrated that radiation, given as fractionated medium-dose regimens, can curtail some of the CAF-mediated inhibiting effects on DC functions. However, the radiation-induced effects were not observed when CAFs were irradiated with a single high-dose. These outcomes suggest that only certain radiation regimens may be able to modify favorably the inherent immunosuppressive functions of CAFs on DCs. The rationale behind these observations is still unknown, and the results presented in this study should also be confirmed in more complex *in vivo* models. Understanding the impact of IR on the multifactorial components in the TME will bring us closer to the ultimate goal of using radiotherapy effectively as an immunological adjuvant in the clinics.

## Data Availability Statement

The original contributions presented in the study are included in the article/**Supplementary Material**. Further inquiries can be directed to the corresponding author.

## Ethics Statement

The Regional Ethical Committee of Northern Norway has approved the use of human material included in this study (REK Nord 2014/401; 2016/714; 2016/2307) and all patients provided informed written consent. 

## Author Contributions

RB, ST, KL, and TH have been implicated in experimental work. TH established radiation protocols and conducted protocols for cell irradiation. RB, IM-Z, ST, and TH carried out evaluation and interpretation of data and drafted the manuscript. RB and IM-Z had the main role in the conception and design of the work. IM-Z has been the main coordinator of the study. All authors contributed to the article and approved the submitted version.

## Funding

This work was supported by the Norwegian Regional Health Authorities under Grant (HNF1373-17 to RB and HNF1423-18 to TH); The Norwegian Cancer Society and the Aakre Foundation at UiT. Publication charges for this article have been funded by a grant from the publication fund at UiT, The Arctic University of Norway.

## Conflict of Interest

The authors declare that the research was conducted in the absence of any commercial or financial relationships that could be construed as a potential conflict of interest.

## Supplementary Material

Supplementary Figure 1 Uncropped scans of Western blots found in **Figure 6**.

Supplementary Figure 2 Uncropped scans of Western blots found in **Figure 7**.

Supplementary Figure 3 Effects of DCs maturation cocktail on CAF-mediated secretion of IL-10 and IL-12. The release of IL-10 and IL-12 from CAFs was measured by ELISA in three different donors stimulated with IL-6, TNF-α, IL-1β, and PGE2, for 48 hrs.

Supplementary Figure 4 Representative micrographs of uptake of FITC-Dextran by immature-DCs previously exposed to conditioned medium from irradiated or non-irradiated CAFs (Two different CAF donors) or in co-culture (CAF/DC).

## Abbreviations

α-SMA, smooth muscle α-actin; CAFs, Cancer-associated fibroblasts; CAF-CM, Cancer-associated fibroblast-conditioned medium; DAMPs, Damage-associated molecular patterns; DCs, Dendritic cells; FAP-1, Fibroblast activation protein 1; GAPDH, Glyceraldehyde-3-phosphate dehydrogenase; GM-CSF, Granulocyte-macrophage colony-stimulating factor; Gy, Gray; HMGB1, High motility group box 1; iDCs, Immature dendritic cells; ICD, Immunogenic cell death; IR, Ionizing radiation; mDCs, Mature dendritic cells; NFκB, Nuclear factor kappa-light-chain-enhancer of activated B cells; NSCLC, Non-small cell lung cancer; PBMC, Peripheral blood mononuclear cells; PGE2, Prostaglandin E2; PI, Propidium iodide; RT, Radiotherapy; STAT3, Signal transducer and activator of transcription 3; TDO2, tryptophan 2,3-dioxygenase; TGF-β, Transforming growth factor-beta; TME, Tumor microenvironment; TNF-α, Tumor necrosis factor-alpha; TSLP, Thymic stromal lymphopoietin; UNN, University Hospital of Northern Norway; VEGF, Vascular endothelial growth factor.
