# Supplementary figures and images for: Ionizing Radiation Curtails Immunosuppressive Effects From Cancer-Associated Fibroblasts on Dendritic Cells
Source: Front Immunol. 2021 Jun 9;12:662594. doi: 10.3389/fimmu.2021.662594 (PMC8221608; doi:10.3389/fimmu.2021.662594)

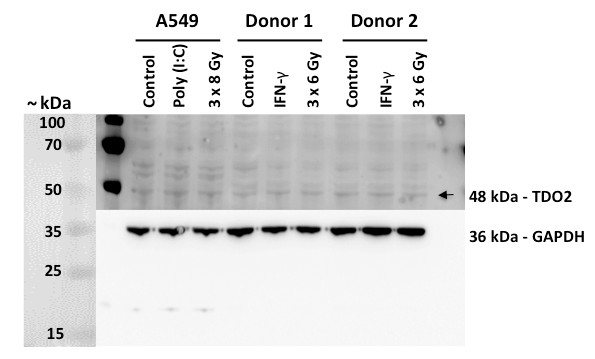

Supplement: Supplementary Figure 2 — Uncropped scans of Western blots found in Figure 7 . [file Image_2.tif]
